# Leveraging User Comments for the Construction of Recycled Water Infrastructure—Evidence from an Eye-Tracking Experiment

**DOI:** 10.3390/bs13010029

**Published:** 2022-12-28

**Authors:** Mengjie Zhang, Caixia Hou, Mengmeng Zhang, Jiachen Niu, Yu Lai, Hanliang Fu

**Affiliations:** 1 School of Management, Xi’an University of Architecture and Technology, Xi’an 710055, China; 2 Laboratory of Neuromanagement in Engineering, Xi’an University of Architecture and Technology, Xi’an 710055, China

**Keywords:** user comments, recycled water infrastructure, recycled water, purchase intention, eye tracking experiments

## Abstract

Building sufficient recycled water infrastructure is an effective way to solve problems related to water shortages and environmental degradation, and is of great strategic significance for saving resources, protecting the ecological environment, and promoting sustainable social and economic development. Although recycled water is environmentally friendly, the public is still skeptical about its use, which has led to the failure of a large number of recycled water infrastructure investments; therefore, increasing the public’s willingness to re-use is critical for the construction of recycled water infrastructure. To identify the influence mechanism of user comments on public re-use behaviors, we conducted an eye-tracking experiment in China. The results demonstrated that (1) perceived usefulness, perceived quality, and perceived risk have significant impacts on the public’s willingness to buy; (2) user reviews can enhance the public’s perceived usefulness of recycled products and increase their willingness to buy; and (3) in the process of consumption, the public tends to pay attention to negative reviews, where user reviews alter the perceived risks and perceived prices of recycled products, thereby affecting the willingness to buy of consumers. This study provides a scientific reference for the construction of recycled water infrastructure and the further promotion of recycled water.

## 1. Introduction

Water resources are the most significant global resource facing environmental issues in the 21st century; it is expected that, by 2050, at least one-quarter of the world’s population will be affected by freshwater shortages. In recent years, the acceleration of industrialization and rapid population growth have led to an increasingly prominent contradiction between water supply and demand [1,2] and, at the same time, the discharge of urban domestic sewage and industrial wastewater has also surged, leading to serious environmental problems and health hazards [3]. Water resources are at the core of national economic security, and in July 2021, China’s National Development and Reform Commission issued the 14th Five-Year Plan for the Development of the Circular Economy, which highlighted the importance of vigorously developing the circular economy and promoting resource conservation and intensive recycling [4]. This requires adequate recycled water infrastructure, a shift from a one-way, straight-line process of “resource–product–waste” to a feedback cycle of “resource–product–waste–renewable resources” [5]. Although recycled products are considered environmentally friendly by consumers and are generally positively evaluated, consumers do not like to use them [6,7]. Recycled water is also regarded as repugnant and ostracized by the public as one of the recycled products; for example, San Diego County’s colossal investment in recycled water infrastructure failed in the 1990s due to massive public protests. Similarly, Australia’s recycled water infrastructure failed, as more than 60% of the public voted against it [8]. It can be seen that technological breakthroughs do not mean the successful implementation of recycled water reuse projects. The successful implementation of recycled water reuse must reasonably and effectively enhance the public’s willingness to reuse. In China, although recycled water has not yet triggered massive opposition, scholars have found that the public has deep-rooted stereotypes against recycled water [9], and even the willingness to consume agricultural products irrigated with recycled water is low [10]. Building sufficient recycled water infrastructure is a critical way to solve the problem of water shortage and water pollution in the process of social and economic development [11]. To avoid the failure of recycled water investments due to public resistance, consumers must be guided to use recycled water actively.

The primary determinant of low public acceptance of recycled water is the “disgust factor”, which can be defined as an aversion or psychological reaction [12]. Wester et al. suggest that the widespread adverse public response to recycled water is mainly due to aversion, especially pathogen aversion [13]. In other words, public concerns about pathogens can be considered the primary driver of emotional reactions to recycled water, which determines the acceptance of water reuse and other cognitive factors. The success of Windhoek’s recycled water for drinking is partly because it meets extremely high specifications and security and health risk issues have been adequately addressed [14]. In contrast, Rozin et al. argue that scientific assurance of recycled water quality and safety does not eliminate the perception of contamination due to “mental contagion”, meaning that advances in water purification technology alone do not automatically guarantee public acceptance [15]. However, public perceptions are shaped by social processes involved in water development, including people’s experiences and the extent to which society discloses information related to recycled water [16]. Therefore, it is crucial to analyze public perceptions of recycled water from an informational perspective, which can provide a more comprehensive understanding of the factors that contribute to public resistance to recycled water reuse.

User reviews mainly refer to the reviews about products made by consumers after purchasing or using them. Studies have shown that the information revealed by user reviews affects the public’s cognitive behavior [17,18], and consumers tend to pay more attention to user reviews for products for which it is difficult to obtain product quality information [19]. At present, consumers have limited channels to understand recycled water, and user reviews have become the most direct way for consumers to understand recycled water and evaluate its quality. Relevant studies have considered product characteristics and product information when analyzing the public’s willingness to use recycled water [20]. Still, few studies have explored the impact of user comments on the public’s willingness to reuse water. Hou et al. have confirmed that user comments play an essential role in the construction of recycled water infrastructure [21], but the mechanism by which user comments affect the public’s recycled water use behavior needs to be further clarified. Therefore, this study considers user reviews and analyzes the effect of user comments on the construction of recycled water infrastructure, which is conducive to the promotion of recycled water and the construction of recycled water infrastructure and, ultimately, sustainable social and economic development.

The purchasing process of consumers involves complex psychological activities [22]. By reviewing the literature, it was found that most previous studies have used questionnaire surveys or text mining to obtain research data [23], through which it isn’t easy to analyze the thinking process underlying individual decision-making comprehensively. The eye–mind hypothesis states that fixation indicates thinking and information extraction and that the eye can genuinely reflect an individual’s cognitive processes. The longer the fixed time, the deeper the degree of cognitive processing [24]. When consumers look at review information, they are performing cognitive processing on the review content. Eye-tracking technology can capture and record the eye movement process of consumers browsing user reviews to form objective data, providing an effective way to analyze the processing of information by consumers [25]. Therefore, in this study, we simulate a recycled water purchase scenario, and conduct eye-tracking experiments to obtain research data and analyze the psychological mechanism underlying the process of public consumption more objectively, in order to put forward reasonable suggestions for the construction of recycled water infrastructure to guide practice. Specifically, we aim to answer the following two questions:

(1) What affects the public’s willingness to buy recycled water?
(2) How do user reviews affect a consumer’s willingness to accept?

## 2. Theoretical Background and Research Assumptions

The American scholar Davis (1986) has proposed the Technology Acceptance Model (TAM) to analyze the influence of an individual’s perceptual and affective factors on the acceptance of information technology [26]. The core idea is that a user’s willingness to use information technology is influenced by a combination of two factors: perceived usefulness and perceived ease of use [27]. Perceived ease of use reflects how easy or difficult consumers perceive information systems to be to operate, whereas perceived usefulness refers to the fact that the more users subjectively approve of information systems and technology products, the more likely they are to accept them. The TAM theoretical model is, to some extent, effective in explaining the acceptance behavior of individuals regarding technology products. However, people’s choices are becoming more and more diverse. From the consumer’s point of view, they will consider not only the usefulness and ease of use but also the cost and benefits of the technology, which is a limitation of the technology acceptance model in explaining consumer purchase behavior. Based on this, Kim et al. (2007) have proposed the Value-based Adoption Model (VAM), the core idea of which is that a user’s willingness to use a technology (e.g., mobile internet) is influenced by a combination of payoffs and benefits [28]; that is, the user’s perceived value. The combination of the TAM and VAM models can consider both technology and consumer perceived value, which provide essential insights to explain public behaviors, such as those related to recycled water use.

According to the TAM theory, an individual’s acceptance of recycled water is influenced by his perceived usefulness of sewage treatment technology and recycled water. Recycled water from sewage treatment can increase revenue and reduce expenditure, reduce sewage discharge, effectively relieve the pressure of water shortage and environmental pollution, and realize a virtuous cycle of water resources [18]. Recycled water is often perceived as “green” and “environmentally friendly” by consumers [29], and individuals who perceive recycled water to be beneficial to the environment tend to be more willing to use recycled water for non-potable purposes [30]. Liu et al. think that individuals’ environmental value of recycled water reuse will directly affect their attitude [31]. However, some scholars have found that people prefer to use recycled water treated by natural water treatment processes (e.g., treated effluent discharged into aquifers or reservoirs), because they believe that discharging to aquifers can further purify treated wastewater, whereas those who are concerned about the potential environmental impact of recycled water are more in favor of replenishing reservoirs to avoid possible pollution of rivers and aquifers [15]. We speculate that this may be related to distrust of wastewater treatment technologies to solve water problems. An earlier study found that trust in technology was associated with greater acceptance of drinking recycled water [32], so we hypothesize that the higher the public’s perceived usefulness of wastewater treatment technology to solve water problems, the more likely they may be to reuse. Based on this, this paper puts forward the following hypotheses to be verified:

**H1:**  *Consumers’ perceived usefulness of sewage treatment technology and recycled water reuse positively influences a consumers’ willingness to purchase recycled water*.

According to the VAM model, consumers will make a comprehensive assessment of the benefits and the costs when purchasing recycled water and make a purchase decision by comparing the benefits to the costs; in other words, consumers will evaluate the quality and cost of using recycled water. Chi et al. found that price was a crucial determinant in purchasing of recycled production by females when they studied consumer perceptions of its value [33]. Price is the monetary cost that consumers must pay to use recycled water. Garcia–Cuerva et al. found that residents would accept recycled water for reuse to save money on water bills when the price of recycled water was significantly lower than tap water [34]. In contrast, Hou et al. found that residents’ willingness to use was highest when it was slightly lower than the price of tap water, rather than significantly lower than tap water [21]. Therefore, this study hypothesizes that public perceptions of recycled water prices significantly affect their willingness to use. Based on this, we propose the following hypotheses:

**H2:**  *The perceived price of recycled water negatively influences a consumer’s willingness to purchase recycled water*.

As for the quality and risk factors of recycled water, Tortajada and Ong concluded that the primary constraint to the implementation of recycled water reuse projects is due to public concerns about the health hazards and environmental impacts of recycled water reuse [35]. Consumers often perceive recycled products as inferior quality to new conventional products, as well as potentially presenting safety risks [36]. Matsumoto et al. have found that Japanese consumers were less knowledgeable regarding remanufactured vehicles and tended to perceive them as providing lower benefits and higher risks, especially regarding quality risks [37]. Similarly, Ong pointed out that there are still shortcomings in wastewater treatment technologies when dealing with some emerging biological and chemical molecules [38], which may lead to recycled water containing harmful microbial and chemical residues that pose a potential threat to human health and ecology [39]. This this raises public doubts about the desirability of treating wastewater and the quality of recycled water [40]. Based on this, we propose the following hypotheses:

**H3:**  *The perceived quality of recycled water positively affects a consumer’s willingness to purchase recycled water*.

**H4:**  *The perceived risk of recycled water negatively influences a consumer’s willingness to purchase recycled water*.

User comments are an essential information source for the public to understand recycled water, as they reflect how others feel about its use. The Elaboration Likelihood Model (ELM) suggests that individuals carefully and systematically analyze the information they receive and consider whether to accept the opinions, thus leading to changes in their attitudes [41]. The information revealed by reviews affects the public decision-making behavior [23]. After viewing user reviews, consumers cognitively process the content of reviews and make subjective judgments about them at a psychological level; this psychological dynamic is the key to determining their final purchase behavior [42]. In other words, consumers will assess the quality of the recycled water through user reviews, then decide whether to purchase it or not. Based on the above, we propose the following hypotheses:

**H5a:**  *User comments indirectly affect consumer purchase intentions by influencing perceived usefulness*.

**H5b:**  *User comments indirectly influence consumer purchase intention by affecting perceived quality*.

**H5c:**  *User comments indirectly affect consumer willingness to purchase by affecting perceived price*.

**H5d:**  *User comments indirectly affect consumer willingness to buy by affecting perceived risk*.

**Figure 1:**
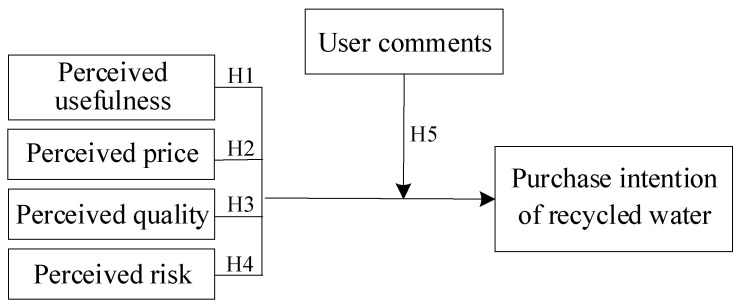
Research model of public recycled water reuse behavior.

Based on the above theoretical background and research hypothesis, we sought to determine the public’s recycled water reuse behavior from the consumers’ perspective (Figure 1). We use eye-movement behavior indicators to measure the public’s willingness to purchase recycled water and select eye-movement gaze indicators as items of user comments to measure their impact on the public’s willingness to purchase recycled water. Further, we measured public perceptions of recycled water using a scale containing the perceived usefulness, perceived price, perceived quality, and perceived risk.

## 3. Materials and Methods

Eye-tracking technology can capture video images of the eyes of experimental participants and record consumer behaviors to form objective indicators [43]. To more realistically and objectively analyze the impact of user comments on the public’s recycled water reuse behavior, we conducted an eye-tracking experiment to record the cognitive processing of user comments and to record the behavior of participants. The eye-tracking device used in this study was a Tobii Pro Fusion, with a sampling rate of 250 Hz.

### 3.1. Design of Stimulus Materials

**Figure 2:**
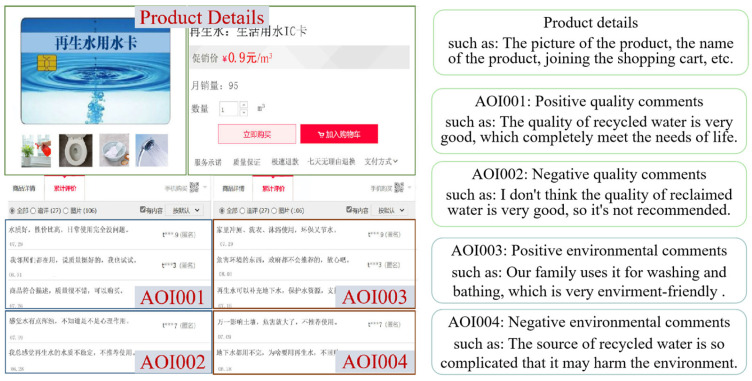
Stimulus picture.

User comments are the content of the user evaluation of recycled water after using it and are an essential source of information for consumers. Safe quality is a prerequisite for the further promotion of recycled water. In studies conducted around the world on the public acceptance of recycled water reuse, it has been found that the public is generally concerned or worried about the quality of recycled water, which leads to their reluctance to use it [44]. Recycled water, as a particular environmentally friendly commodity, can significantly alleviate regional water shortages and enable water recycling [45]. Some scholars have found that individuals who are concerned about future water supplies and the state of the water environment conditions may be more likely to accept recycled water [46]. Therefore, in this study, user reviews characterizing the quality of recycled water and the impact of recycled water on the environment were selected as the content of the experimental material, designed based on Taobao product detail pages, to enhance the immersion and experience of the participants. Considering that negative reviews have a more significant impact on consumer decision-making behaviors, we used three positive and two negative reviews and adopted a within-group experimental design of 2 (good quality vs. bad environmental reviews) × 2 (good environmental reviews vs. bad environmental reviews). We set the reviews into four interest areas according to the polarity of the reviews (Figure 2). The top-left area of the material included positive quality reviews (AOI001), the bottom-left area was negative quality reviews (AOI002), the top-right area was positive environmental reviews (AOI003), and the bottom-right area was negative environmental reviews (AOI004). All comments had similar word counts. Considering that the length of gaze may also be due to the subject’s difficulty understanding stimuli, we screened the content of the comments and selected comments with clear and concise language expressions. The subjects have sufficient time to browse through the interface information for each recycled water product in the experiment. The average fixation time was calculated as the average duration of all gaze points in the interest area, a critical eye-movement indicator characterizing the cognitive process of consumers, which has been used widely in consumer behavior research. In this study, the average fixation time in the area of interest was selected as an eye-movement indicator of consumer cognitive processing and as the observed variable for user comments to analyze further the mechanism of its influence on the willingness to purchase recycled water.

### 3.2. Experimental Process

**Figure 3:**
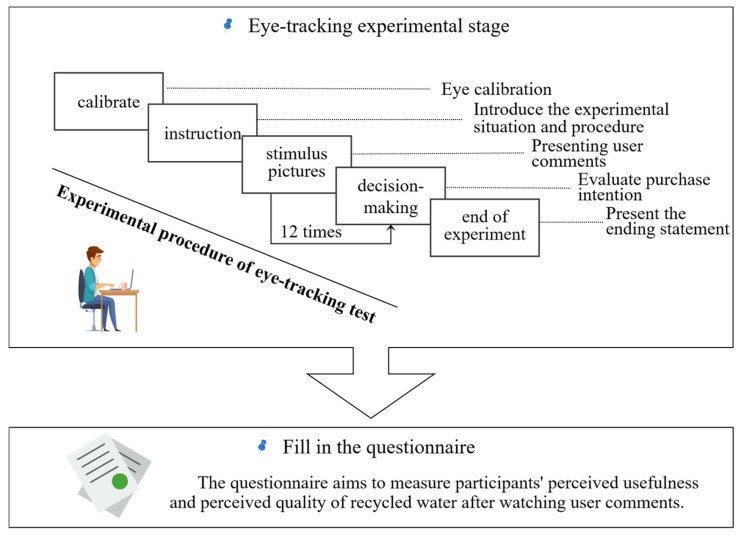
Experimental procedure.

The experiment was completed in the neuroengineering management laboratory of Xi’an University of Architecture and Technology, with good indoor air quality and appropriate light and temperature. A total of 13 participants took part in the pre-experiment. In the formal experiment, 7 people were excluded due to low eye-movement data collection rates, and data from 94 experimental participants (51 males and 43 females) were used. All experimental participants had normal or corrected visual acuity. In the preparation phase of the experiment, the main subjects informed the participants about the precautions and operational methods. In the formal experiment, after calibrating the subject’s eyes, the screen would present experimental instructions to guide the participants into the specific practical situation: “Assuming that your home has been renovated with recycled water pipes, you can use recycled water by simply recharging the card (the card is free), and the tap water can also use normally”. The next screen showed the details of the recycled water product, which mainly contained user comments (Figure 2), and the participant was given plenty of time to browse. After browsing, the participant pressed the space bar and entered the shopping decision screen, where the participant was asked to assess their willingness to buy recycled water, rated from 1 (very reluctant to buy) to 7 (very willing to buy), by pressing the relevant number on the keyboard. After browsing all the pages, the experiment ended. Subjects were required to report their perception of recycled water during the shopping process and fill out a questionnaire. The specific experimental flow is shown in Figure 3.

### 3.3. Design of Questionnaire

To explore the influence of user comments on public cognitive behavior, we used a questionnaire to collect data on consumer cognitive behavior. The final scale was obtained by iteratively revising the results of this study based on the pre-study. The questionnaire was divided into two parts: one part contained basic information about the respondent’s age, gender, education level, and income level; the other part contained measured variables, such as the perceived usefulness, perceived price, perceived quality, and perceived risk of recycled water (Table 1). The questionnaire items were scored subjectively by individuals on a seven-point Likert scale, with scores from 1 to 7 representing oppose strongly, oppose, somewhat oppose, neutral, somewhat agree, agree, and agree strongly, respectively.

Structural equation modeling is a statistical analysis technique based on an existing causal theory, representing that causal theory with a corresponding system of linear equations. Commonly used modeling methods include two main types: Linear Structural Relations, based on the covariance distance array, and Partial Least Squares Regression, based on the partial least squares path. Compared with the covariance-based structural equation model, PLS-SEM requires a smaller sample size, with a recommended sample size of 30–100, as the PLS method requires a sample size of 10 times the number of construct terms. As eye-tracking experiments are generally limited by the sample size, we chose PLS-SEM as the modeling method. We used the Smart PLS 3.0 software (SmartPLS GmbH; Gewerbering 8, D-22114 Oststeinbek, Germany) to analyze the intrinsic relationship between review information and willingness to purchase recycled water.

## 4. Result

### 4.1. Reliability and Validity Test

To ensure the reliability and validity of the data, we calculated the composite reliability (CR), average variance extracted (AVE), and Cronbach’s alpha values (Table 1). The results demonstrated that Cronbach’s alpha coefficients for all variables were 0.645–0.948, mainly more excellent than the threshold value of 0.7, and the CR was 0.849–0.938, greater than the threshold value of 0.6. Therefore, the experimental scale can be considered to have good reliability.

The validity tests of the scale mainly consisted of convergent validity and discriminant validity tests of the scale. As shown in Table 1, the t-values of factor loadings for all observed variables were statistically significant (*p* < 0.001), indicating good convergent validity. In addition, aggregation and discriminant validity were tested by variance extraction tests. In the variance extraction test, the AVE value of each structural variable in the model requires greater than the square of the correlation coefficient between structural variables (i.e., the square root of AVE is greater than the correlation coefficient between structural variables). The results of the variance extraction test are shown in Table 2, where the square root of AVE on the diagonal can be seen to be greater than the rest of the correlation coefficients in the same column of the peer group.

In addition, we further examined the discriminant validity of the constructs using the Heterotrait–Monotrait Ratio (HTMT), which should generally be less than 0.85; the results indicated that they all met the requirements (Table 3). Therefore, this scale was found to have good convergent and discriminant validity.

### 4.2. Factors Influencing the Public’s Willingness to Use Recycled Water

**Figure 4:**
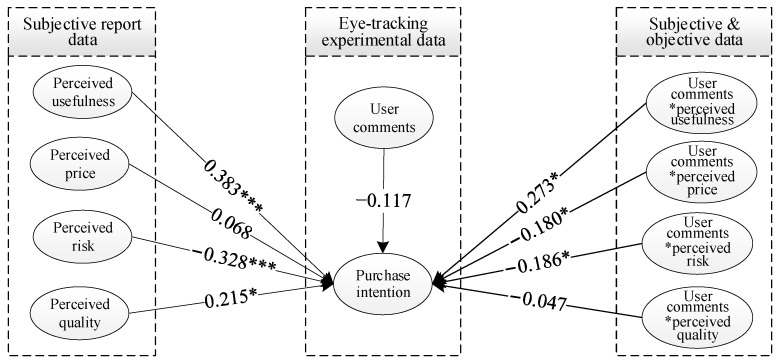
Mechanism of user comments on the public’s willingness to purchase recycled water. Note: * means significant at 0.05 level correlation; *** means significant at 0.001 level correlation.

The model simulation results showed that, in addition to perceived price, perceived usefulness, perceived quality, and perceived risk all affect the recycled water purchase intention (Figure 4). Perceived usefulness had the most significant effect on recycled water purchase intention and was positively correlated with a path coefficient of 0.383, indicating that individuals who think that wastewater treatment technology is useful are more willing to use recycled water to protect the environment and save water resources; therefore, Hypothesis 1 was verified. Secondly, perceived risk negatively affected the public’s willingness to purchase recycled water, with a path coefficient of −0.328, and perceived quality positively affected the public’s willingness to purchase recycled water, with a path coefficient of 0.125. It can be concluded that those who tend to use recycled water believe that its quality is better, and the risk is lower; thus, Hypotheses 3 and 4 were also confirmed. However, there was no statistically significant relationship between perceived price and public willingness to purchase recycled water. Hypothesis 2 was not valid.

### 4.3. Mechanism of User Comments on the Public’s Intention to Purchase Recycled Water

**Figure 5:**
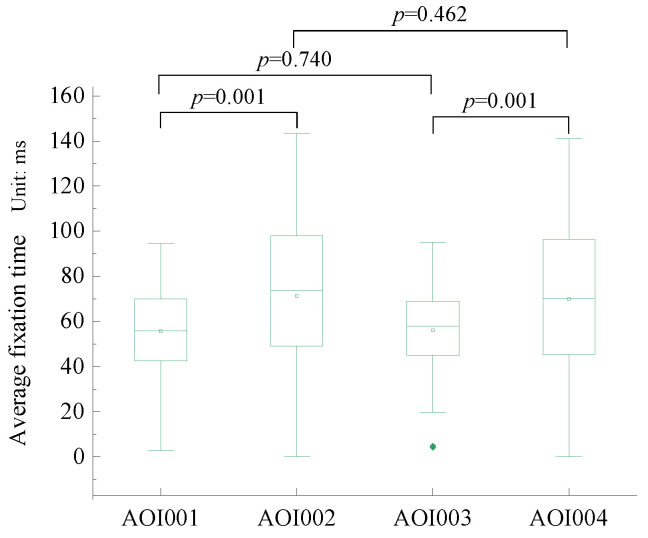
Paired-sample *t*-test for the average fixation duration of the AOI.

To investigate the mechanism of user reviews on the public’s willingness to use recycled water, we first conducted a paired-sample t-test on the average fixation duration of different interest areas to analyze the public’s attention to four types of comments (Figure 5). Considering that the inconsistency between the number of positive and negative reviews led to the data not being comparable, we used the average fixed time on each review for comparison. The results indicated that the mean fixed duration of both positive and negative quality reviews and both positive and negative environmental reviews significantly differed at the significance level of *p* < 0.001 (t = −6.464, *p* = 0.001; and t = −5.349, *p* = 0.001, respectively), which indicates that the public is more concerned about the content of negative reviews than positive reviews in the public recycled water purchase decision process, as well as more concerned about negative impacts caused by the use of recycled water.

**Figure 6:**
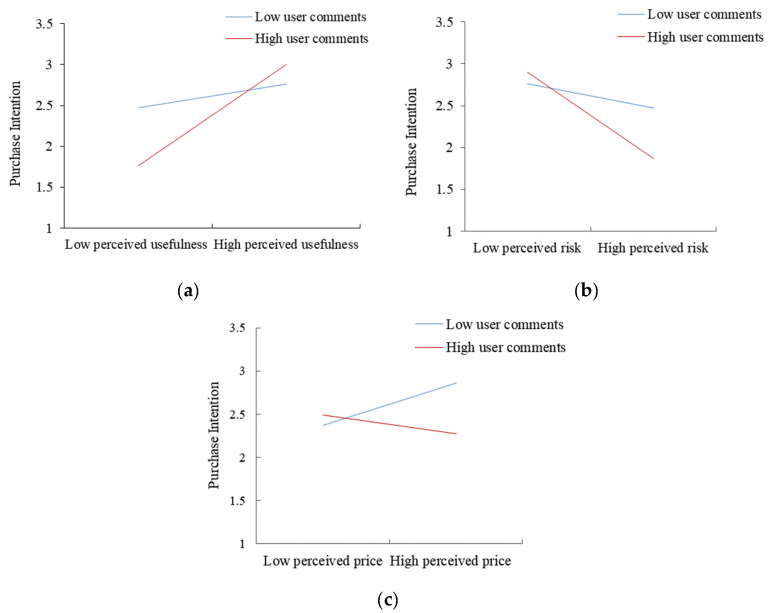
Interaction effects of user reviews with perceived usefulness, perceived risk, and perceived price: (**a**) The moderating effect of user comments on perceived usefulness and purchase intention; (**b**) The moderating effect of user comments on perceived risk and purchase intention; (**c**) The moderating effect of user comments on the perceived price and purchase intention.

The model simulation results indicated that user comments positively moderate the relationship between perceived usefulness and willingness to purchase recycled water, with a path coefficient of 0.237, which was significant at the 0.05 level (Figure 4); therefore, Hypothesis 5 was confirmed. This indicates that user comments indirectly affect the public’s willingness to purchase recycled water by influencing the perceived usefulness of recycled water reuse: the higher the public’s attention to user comments, the more likely it is to stimulate the public’s perceived usefulness of recycled water, thus promoting its willingness to purchase recycled water. The interaction between user comments and the perceived usefulness of recycled water reuse is depicted in Figure 6a. The average attention time of consumers to the comment information was divided into high- and low-information attention groups to analyze the role of perceived usefulness on willingness to purchase. It was found that the positive effect of perceived usefulness and recycled water purchase intention was more substantial for consumers with high user review attention. Therefore, increasing their engagement with recycled water reviews can effectively promote their willingness to purchase it. To encourage the public’s desire to purchase recycled water, policymakers should not only promote the environmental effectiveness of wastewater treatment technology, but also encourage recycled water users to comment on it actively, such that the effectiveness of the policy may be improved.

The results of the SEM indicated that user comments negatively moderate the relationship between perceived risk and willingness to purchase recycled water, with a path coefficient of −0.186, which was significant at the 0.05 level (Figure 4). It can be seen that user comments enhance the public’s perceived risk and, thus, negatively affect the willingness to purchase recycled water. The interaction between user comments and the perceived risk of recycled water reuse is shown in Figure 6b. The average fixation time of the public on the user comments was again divided into high- and low-information concern groups to analyze the moderating effect on the perception of perceived risk and public willingness to purchase recycled water. The negative impact of perceived risk and willingness to purchase recycled water was more substantial for individuals with close attention to user comments and weaker for those with low attention to user comments. This suggests that public awareness to user reviews reinforces the negative effect of perceived risk on willingness to purchase recycled water.

The results of this study demonstrate that user reviews negatively moderate the relationship between perceived price and willingness to purchase recycled water, with a path coefficient of −0.180 and a significant negative interaction at the 0.05 level (Figure 4). Therefore, user comments reduce the public perception of recycled water price and, thus, affect the willingness to purchase recycled water. The interaction between user reviews and the perceived price of recycled water reuse is shown in Figure 6c. The average fixation time of consumers on review information was once more divided into high- and low-information concern groups to analyze the moderating effect of users on perceived price and general desire to purchase recycled water. The relationship between perceived price and public willingness to purchase recycled water was weaker for individuals with great attention to user reviews. For those with low attention to user reviews, the relationship between perceived price and willingness to purchase recycled water was negatively correlated, indicating that public awareness to user reviews weakens the relationship between perceived price and desire to buy.

## 5. Discussion

### 5.1. Factors Influencing the Public’s Willingness to Purchase Recycled Water

Individual behaviors are governed by an individual’s thoughts and perceptions [48]. In this study, we found that perceived usefulness positively influences the public’s willingness to purchase recycled water. Wastewater reuse provides an essential means to alleviate water scarcity and solve environmental pollution-related problems. As a renewable resource product, recycled water has good ecological benefits. Its perceived usefulness refers to the public’s recognition of the environmental benefits and water-saving potential of wastewater reuse. The higher the perceived usefulness, the more the public recognizes the environmental value of recycled water reuse, the more positive their attitude toward recycled water, and the stronger their willingness to purchase it. This result is consistent with the findings of Bulut and Nazli, where perceived usefulness was an essential predictor of consumer willingness to buy green products [49]. It plays a vital role in the consumer purchase decision process [50]. It has also been argued that individual awareness of water conservation and environmental responsibility can effectively promote the willingness to use recycled water [51]. Therefore, policymakers should vigorously promote the positive effects of building recycled water infrastructure on water scarcity and the ecological environment, enhance the public’s perceived usefulness of wastewater reuse and, at the same time, continuously raise the public’s awareness of environmental responsibility through various means.

Perceived quality is a consumer’s subjective judgment of recycled water quality through product information clues. In China, the public is still in the wait-and-see stage regarding recycled water reuse, especially for domestic use. The safety of recycled water quality and whether recycled water reuse can bring health risks to themselves and their families are the first factors that the public considers when thinking about recycled water reuse. The results of this study indicated that perceived quality is significantly and positively correlated with the willingness to purchase recycled water, whereas the perceived risk is negatively correlated with the desire to purchase recycled water. The higher the public recognition of the safety and stability of recycled water quality, the lower the perceived risk of recycled water reuse and the more inclined they are to use recycled water. This is consistent with the findings of Hou et al., where residents’ concerns about the health risks of recycled water reuse were shown to be an essential factor contributing to their opposition to recycled water reuse projects [47]. Of course, research on public concerns about the safety of recycled water does not stop here; Jeffrey and Jefferson have found that people are skeptical about the “reliability of technology” and “Stakeholders including water suppliers, academic researchers, regulators, and general community members”, which led them to perceive higher health risks [52]. In a review of previous studies, Nkhoma et al. have found that numerous scholars cited the perception of safety risks associated with recycled water as a potential influence on the residents’ willingness to accept them [50], which may inversely influence the finding on the public’s desire to use [53].

The results of this study demonstrated that perceived price does not have a significant effect on a consumer’s willingness to purchase recycled water. We discerned several reasons for this result. The perceived price of recycled water was measured according to the questions “recycled water is cost-effective” and “the price of recycled water is reasonable”. First, due to differences in individual characteristics, such as income and living environment, there is a difference in the measure of the cost-effectiveness of recycled water among individuals, which may lead to some individuals not perceiving recycled water to be cost-effective, leading to the absence of a direct relationship between perceived price and purchase intention. Second, the lack of a general understanding of water pricing mechanisms among some individuals has led to a more ambiguous public knowledge of the issue of recycled water prices, and it may be not easy to associate people’s perceptions of prices directly with their willingness to purchase.

### 5.2. Mechanism of User Comments on the Public’s Willingness to Use Recycled Water

The study results showed that the public is more concerned about negative comments than positive comments about the quality and the environment. Some scholars found that the effectiveness of information depends on the initial attitude of the target audience [18], and some scholars found that the public holds negative implicit attitudes toward recycled water [54]. On the one hand, due to the negative attitude toward recycled water, the public may pay more attention to the content of negative reviews of recycled water during the consumption process to corroborate their opinions. In addition, from the consumer’s point of view, the public will evaluate the health risks and environmental impacts of recycled water in the purchase process to avoid risks and make decisions after considering the pros and cons. Therefore, the public may pay more attention to negative comments and process them more cognitively.

Personal decision theory suggests that consumers evaluate the possible benefits and risks of a product [55]. In this study, we found that user reviews positively moderated the relationship between perceived usefulness and the public’s willingness to purchase recycled water. Attention to user reviews increased the approval of recycled water reuse by individuals, thus strengthening their desire to reuse recycled water. This is consistent with the study of Pretner et al. [56], who argued that providing environmental information to consumers with insufficient knowledge of recycled products can help to reduce their inner suspicion and increase their perception of the environmental benefits of recycled products. User reviews, which detail real experiences with recycled water, are an essential source of information for consumers to understand recycled water; therefore, user reviews can effectively improve the public’s perceived usefulness of recycled water. It has also been found that recycled water disclosure has an “amplifier” effect on the relationship between awareness of water conservation and willingness to reuse recycled water [57]. Therefore, disclosing the environmental status and benefits of recycled water to increase the public’s perceived usefulness can effectively improve the public’s willingness to use recycled water, thus promoting the construction of recycled water infrastructure. Price’s experiment found that the public’s support for recycled water would increase after providing information about recycled water, but at the same time, when providing users with information about the relative risks and benefits of recycled water, the support for recycled water would increase even more [58]. Moreover, he also found that the public’s initial attitude towards recycled water would regulate the public’s response to the information. Therefore, in the process of information disclosure, we should not only pay attention to the design of more detailed information but also pay attention to the initial attitude of the public towards recycled water and carry out information publicity differently to maximize the effectiveness of information.

In this study, we observed a significant negative interaction between the two dimensions of user reviews and the perceived risk of recycled water reuse; that is, the negative effect of perceived risk on the public’s willingness to purchase increased with an increase in user review concern. Recycled water is being promoted in China, but public knowledge and awareness regarding recycled water are still relatively low [59]. Some studies have found that consumers with less existing knowledge are more likely to be driven by the emotional content of reviews [60]. Moreover, negative product information triggers a more robust emotional response in consumers compared to positive product information [61]. To assess the quality of recycled water, the public may rely more on the reviews of others. As the public is more inclined to look at negative reviews, the cognitive processing of negative thoughts is higher; individuals may even amplify their perception of the risk to their safety, which may lead to higher attention being paid to the content of reviews negatively moderating the relationship between perceived risk and purchase intention.

We also found a significant negative interaction between the two dimensions of user reviews and perceived price; that is, the perceived price was negatively correlated with the public’s willingness to purchase recycled water for individuals with great attention to user reviews and positively correlated with the public’s willingness to purchase recycled water for those with low concentration to user reviews. Perceived price refers to a consumer’s psychological feelings about price. It has been found that a consumer’s perceived value is highly correlated with their perceived sacrifice. When perceived quality is greater than the perceived sacrifice, perceived value is positive, and more consumers tend to buy the product [62]. As residents have negative implicit attitudes toward recycled water [63], and negative comments convey the risks associated with recycled water reuse to consumers, this increases the public’s perception of the health risks associated with recycled water reuse, resulting in the perceived payoff being more significant than the perceived benefit and the perception that using recycled water is not cost-effective, which may be the reason for the negative correlation between perceived price and purchase intention in the high user comments group. In contrast, in the low-concern group, individuals may be less influenced by negative comments, and those who perceive recycled water as cost-effective are more willing to use recycled water. Therefore, the most important task for managers is to ensure the safety of recycled water and improve the public’s perceived quality through various means to reduce public concerns and safety worries about recycled water, thereby reducing negative public comments at the root.

Due to technical and researcher capacity limitations, we were unable to specify which specific types of comments caused the change in public perception and willingness to use, but this gave us great motivation to continue to try to clarify the effects of different types of user comments in future studies on the role of user comments in the promotion of recycled water reuse.

## 6. Conclusions and Recommendations

The construction of recycled water infrastructure is strategically important to conserve resources, protect the environment, and achieve sustainable socio-economic development. In this study, we conducted an eye-tracking experiment focused on public recycled water purchase behavior to explore the mechanism by which user reviews affect public recycled water reuse behavior by simulating a real purchase scenario. We obtained the following main conclusions: (1) perceived usefulness, perceived quality, and perceived risk all have significant effects on public purchase intentions, with perceived usefulness having the greatest effect; (2) the public is inclined to pay more attention to negative reviews, and user reviews presented the largest moderating effect between perceived usefulness and purchase intention, followed by their moderating effect on perceived risk and purchase intention. Furthermore, user reviews also had a significant moderating effect on perceived price and purchase intention.

Based on the above findings, we propose the following recommendations to improve the public’s willingness to purchase recycled water and promote the construction of recycled water infrastructure: First, we found that perceived usefulness, perceived risk, and perceived quality directly affect the public’s willingness to purchase. Therefore, managers should vigorously promote the current state of water resources and the water environment through various channels to increase the public’s sense of environmental urgency, as well as increase the publicity of the environmental benefits of building recycled water infrastructure and its necessity, to enhance the public’s perceived usefulness of recycled water. At the same time, the quality and safety of recycled water should be guaranteed, and information on recycled water quality standards and production should be disclosed to the public, thus weakening public concerns and aversions regarding recycled water. Companies can also promote experiential activities, such as product trials, which can help consumers to understand the value of recycled water better and weaken their worries and doubts about its quality and safety, thus promoting the public’s willingness to accept it. Second, we observed that user reviews could enhance public willingness to accept by strengthening perceived usefulness. Therefore, managers should encourage users to share their feelings and experiences regarding the use of recycled water, especially positive evaluations of recycled water reuse in the surrounding environment. Again, we found that the public is biased in that they generally pay more attention to negative reviews and that user reviews negatively moderate the relationships between perceived risk, perceived price, and public willingness to purchase. Therefore, production technology should be continuously improved to avoid the health risks associated with recycled water. Managers should strictly regulate the quality of the recycled water while using various media and adopting various forms (e.g., thematic education, knowledge competitions, visits, community consultation, and so on) to disseminate specific and targeted information about recycled water—including quality, quality standards, regulatory systems, and so on—to the public, to alter the negative public perception of recycled water. Reducing negative consumer perceptions of recycled water is expected to have the effect of increasing the public’s willingness to reuse recycled water and promote the construction of recycled water infrastructure.

## Figures and Tables

**Table 1:** Reliability and validity indicator results.

Latent Variable	Perceived Usefulness			Perceived Quality			Perceived Price		Perceived Risk		User Comments	Purchase Intention
Observed variables	Wastewater reuse can reduce water scarcity.	Wastewater treatment technology can reduce environmental pollution.	Wastewater reuse can realize the recycling of water resources	The quality of recycled water is reliable.	The quality of recycled water is entirely sufficient for domestic needs.	The quality of recycled water is stable.	The price of recycled water is reasonable.	Recycled water is cost-effective.	Using recycled water can affect my health and that of my family.	I’m concerned about the safety of recycled water.		
Factor loading	0.866	0.819	0.689	0.862	0.792	0.712	0.852	0.866	0.897	0.867	1.000	1.000
CR	0.872			0.836			0.849		0.875		0.968	1.000
AVE	0.696			0.632			0.738		0.938		0.968	1.000
Cronbach’s alpha	0.779			0.710			0.645		0.715		0.948	1.000
Citation	[31]			[47]			[34]		[31,47]			

**Table 2:** Average variance extraction.

	Perceived Price	Perceived Usefulness	Perceived Quality	Perceived Risk	User Comments	Purchase Intention
**P** **erceived price**	0.859					
**P** **erceived usefulness**	0.425	0.834				
**P** **erceived quality**	0.388	0.430	0.795			
**Perceived risk**	−0.220	−0.151	−0.565	0.882		
**U** **ser comments**	−0.019	0.106	0.078	0.055	0.969	
**Purchase intention**	0.434	0.481	0.555	−0.490	−0.079	1.000

**Table 3:** Average variance extracted.

	Perceived Price	Perceived Usefulness	Perceived Quality	Perceived Risk	User Comments	Purchase Intention
**Perceived price**						
**Perceived usefulness**	0.592					
**Perceived quality**	0.600	0.575				
**Perceived risk**	0.328	0.232	0.778			
**User comments**	0.023	0.170	0.186	0.093		
**Purchase intention**	0.540	0.536	0.644	0.578	0.097	

## Data Availability

The supplemental raw data used to support the findings of this study are available upon request from the corresponding author. All the data contained in this study can be obtained upon request from the corresponding author.
